# Evaluation of the retinal, choroidal, and nerve fiber layer thickness changes in patients with toxic anterior segment syndrome

**DOI:** 10.1007/s00417-014-2880-3

**Published:** 2014-12-03

**Authors:** Nir Sorkin, Dafna Goldenberg, Amir Rosenblatt, Gabi Shemesh

**Affiliations:** Department of Ophthalmology, Tel Aviv Medical Center and the Sackler Faculty of Medicine, Tel Aviv University, 6 Weizmann Street, Tel Aviv, 6423906 Israel

**Keywords:** Choroid, Enhanced depth imaging, Optical coherence tomography, Retina, Toxic anterior segment syndrome

## Abstract

**Purpose:**

To evaluate changes in choroidal, retinal, and nerve fiber layer (NFL) thickness following toxic anterior segment syndrome (TASS).

**Methods:**

Macular and peripapillary choroidal thickness was measured using enhanced depth imaging (EDI) optical coherence tomography (OCT) on the day of the diagnosis and on three follow-up exams (months 1 to 4). A similar OCT analysis of central retinal and NFL thickness was performed.

**Results:**

Thirteen TASS patients were included. Average age was 72.8 ± 8.7 years. Macular choroidal thickness in the superior, subfoveal, and nasal macula in the study eye was larger than the control eye and decreased at months 2–4. This was statistically significant only for the superior macula (p = 0.004). Peripapillary choroidal thickness was larger in the study eye at baseline compared with the control eye—significantly so in the nasal (p = 0.026) and inferior (p = 0.033) locations. Peripapillary choroidal thickness peaked at the baseline or 1st month exam and decreased thereafter. Retinal thickness increased significantly with time, peaking at the 2nd month and decreasing thereafter. No changes were found in the NFL.

**Conclusions:**

TASS may have a transient effect on the choroid. Changes in retinal thickness are probably a normal transient postoperative response and not a result of TASS.

## Introduction

Cataract surgery is one of the most common surgeries performed worldwide, with a high rate of success. Toxic anterior segment syndrome (TASS), a term coined by Monson et al. in 1992 [1], is a postoperative complication reported in increasing numbers. It is a sterile, postoperative inflammation occurring early after anterior segment surgery, generally appearing within 12–48 h [2]. It may result in damage to various intraocular tissues.

Typically, TASS presents acutely, but rarely can have a delayed onset. Symptoms include blurry vision, discomfort, and minimal pain. Signs include eye redness, fibrin formation with or without hypopyon, inflammatory membranes, and diffuse corneal edema secondary to endothelial damage. In addition, potential damage to the iris and trabecular meshwork may occur. The inflammation is limited to the anterior segment. TASS can present sporadically or in outbreaks [3], possible causes are numerous [4], but in many of the cases the cause is not identified. The outcome of topical steroidal drops and close monitoring in the management of TASS is excellent [5]. However, severe complications, such as irreversible visual loss, permanent endothelial damage, corneal decompensation, a permanently dilated pupil, or glaucoma due to permanent damage to the trabecular meshwork, may occur [2, 6].

TASS has many similarities to other postoperative inflammatory processes, such as uveitis and endophthalmitis [7, 8]. However, it can be distinguished from endophthalmitis as it seems to be limited to the anterior segment [2].

In the past, choroidal thickness could not be accurately measured. Recent advancements in optical coherence tomography (OCT) with the use of enhanced depth imaging (EDI) allow more accurate evaluation of choroidal thickness in a reproducible manner, as shown in previous studies [9–11]. In the field of inflammatory eye diseases, this technique has been used to demonstrate changes in choroidal thickness in Vogt-Koyanagi-Harada panuveitis subjects [12]. The inflammatory process seen in TASS influences anterior uveal structures. Our aim was to investigate whether these inflammatory changes have an effect on the posterior uvea and retina and whether subtle choroidal and retinal changes, which cannot be noticed clinically, exist.

## Materials and methods

This prospective study adhered to the tenets of the Declaration of Helsinki and was approved by the institutional review board (IRB) of the Tel Aviv Medical Center. Informed consent was obtained from all patients.

### Participants

Thirteen eyes of 13 subjects, consecutively diagnosed with TASS following cataract surgery, were enrolled.

A diagnosis of TASS was established based on the clinical findings of a postoperative anterior chamber inflammatory response with fibrin formation in either the anterior chamber, the pupillary aperture, or just anterior to the posterior chamber intraocular lens. All subjects had no clinical evidence of posterior segment involvement (no vitreous inflammation and no acute retinal findings on a dilated fundus examination). Following a clinical diagnosis of TASS, a complete ophthalmological examination was performed including visual acuity (VA), slit-lamp examination, Goldmann applanation tonometry, and fundus examination. In addition, a baseline spectral domain OCT (SD-OCT) scan was performed.

Subjects underwent repeat biomicroscopic and SD-OCT examinations, performed on month 1 (first follow-up), month 2 (second follow-up), and a final follow-up exam performed at months 3 or 4 (third follow-up).

### Spectral domain optical coherence tomography

**Fig. 1:**
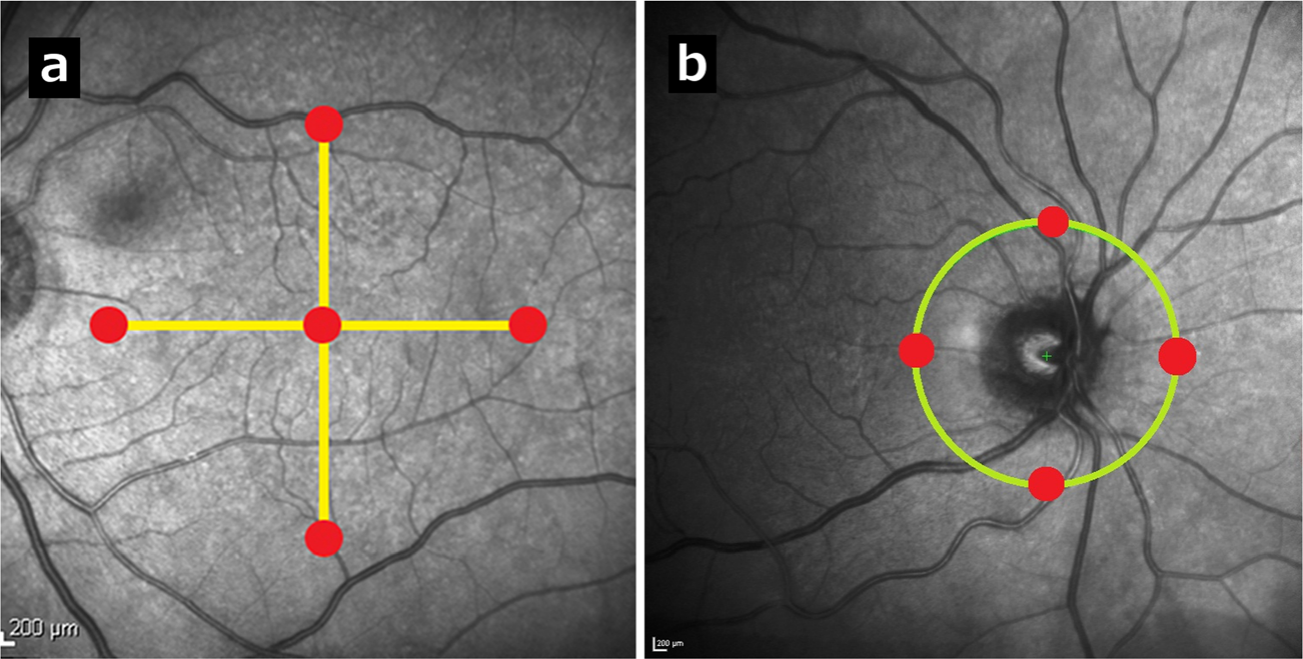
**a.** (Patient 13) A left eye infrared map of enhanced depth imaging (EDI) optical coherence tomography (OCT) foveal measurement locations (red circles) at the foveal center and 2.5 mm nasal, temporal, inferior, and superior to the foveal center. The yellow lines represent the OCT cross-sections. **b.** (Patient 1) A right eye infrared map of EDI-OCT peripapillary measurement locations (red circles) 1.75 mm nasal, temporal, inferior, and superior to the optic disc center. The yellow-green circle represents the OCT cross-section

All participants were examined using Heidelberg Spectralis SD-OCT (Heidelberg Engineering, Heidelberg, Germany), which achieves an axial resolution of 7 μm. The retinas of both eyes were first scanned by a horizontal high-speed raster scanning protocol of a 20°X20° quadrangular area, and central macular thickness (CMT) was obtained. Glaucoma retinal NFL protocol scan was then performed, and inferior, superior, nasal, and temporal NFL thickness measurements were recorded. In addition, in order to demonstrate choroidal thickness changes, EDI technique [10, 11] was used. The choroid was scanned with 20° horizontal and vertical single cross-section EDI scans through the center of the fovea and by a circular EDI NFL scan protocol at the peripapilary region. To ensure high quality and noise reduction, the eye tracking system and averaging technique was employed. Each choroidal scan consisted of 100 averaged OCT frames. Images were converted to white on black grayscale to sharpen the contrast and allow a more accurate measurement. Choroidal thickness was measured by a single investigator, with an SD-OCT software electronic caliper. Choroidal thickness was defined as the vertical distance between the outer margin of the hyper-reflective retinal pigment epithelium layer and the chorioscleral interface. Choroidal thickness was measured at five macular locations: subfoveal (an average of two subfoveal measurements—one from the horizontal and the other from the vertical cross-section) and 2.5 mm nasal, temporal, inferior, and superior to the foveal center (Fig. 1a). Similar measurements were performed in the peripapillary region in four different locations: 1.75 mm superior, temporal, inferior, and nasal to the optic disc center (Fig. 1b).

The follow-up acquisition mode, unique to this SD-OCT device, was used in all follow-up visits and automatically placed follow-up scans in precisely the same anatomic location as previous scans.

Two analyses of OCT measurements were performed: The first compared measurements of the study eye with measurements of the fellow eye, while the second analysis examined measurement changes in the study eye over time.

### Choroidal thickness measurement correction

Previously published data demonstrated that choroidal thickness is influenced by axial length [13]. There is an inverse relationship between axial length and choroidal thickness. Therefore, choroidal thickness measurements were corrected according to the difference in axial length between the study and fellow eye. The correction coefficient used to compensate for axial length differences between the study and fellow eye was based on previously published literature [14]—for every 1 mm difference in axial length, choroidal thickness was reduced by 22 μm.

There was no need for age-related adjustments of choroidal thickness because all comparisons were performed either in the same eye or with the fellow eye of the same patient with no inter-patient comparisons.

### Axial length

Axial length measurements were obtained from the patient's medical records (standard measurements performed prior to a cataract surgery). Measurements were performed using the IOLMaster (Carl Zeiss, Oberkochen, Germany) biometer. In cases of media opacities, interfering with the biometer measurements, axial length was measured using an A-scan 10-MHz transducer with minimal corneal compression and with the use of topical anesthesia. The recorded value was the mean of six reliable measurements.

### Exclusion criteria

Exclusion criteria included cystoid macular edema of any etiology, history of endophthalmitis or other infectious process in the study eye, history of idiopathic or autoimmune-associated uveitis in either eye, history of any posterior segment operation prior to the cataract procedure in either eye, any procedure, but for uneventful cataract surgery, patients receiving steroidal treatment (systemic or topical) prior to the cataract surgery, and media opacities preventing OCT imaging of the retina or choroid.

### Statistical analysis

Data was recorded using Microsoft Excel and analyzed using SPSS version 21 (SPSS Inc., Chicago, IL, USA). Because of the small sample size, non-parametric tests were used. The Friedman test was used to compare overall difference in measurements over time (including baseline exam and the three follow-up exams) or difference between the choroidal thickness measurement locations.

Pairwise comparison of two time-point or measurement locations was analyzed by the Wilcoxon Matched-Pairs Signed-Ranks Test. All tests were two-tailed, and the threshold for statistical significance was defined as a p value <0.05.

## Results

### Patients and procedures

Thirteen participants were included in the study—nine women and four men. The average age was 72.8 ± 8.7 years (range 57–89 years). There were three left study eyes and 10 right study eyes. Two patients had bilateral epiretinal membrane with no evidence of macular edema, and one patient had a history of branch retinal vein occlusion in his control eye with no evidence of macular edema prior to or following surgery. All patients had undergone uneventful cataract extraction surgeries.

Twelve of 13 patients had normal findings at the routine 1-day postoperative exam and have been diagnosed with TASS at the routine 1-week postoperative exam (mean 8.1 days). They did not seek earlier ophthalmologic consult prior to the routine 1-week exam. One patient presented at the clinic 3 days following surgery, with TASS-related complaints and was then diagnosed. All TASS cases resolved under topical steroidal treatment (in 10 of 13 eyes, a mydriatic agent was also administered). One patient had residual posterior iris synechiae, one patient developed pseudophakic CME (diagnosed 8 weeks following TASS diagnosis), and a third patient had both residual synechiae and mild pseudophakic CME (diagnosed 10 weeks following TASS diagnosis). The remaining 10 patients had no stigmata following TASS resolution. Average follow-up times (post TASS diagnosis) for the first, second, and third follow-up exams were: day 10.5 ± 5.6, day 35 ± 7.7, and day 95.2 ± 23.8, accordingly. Follow-up exams included clinical evaluation and OCT exams.

### Macular choroidal thickness measurements

Mean macular choroidal thickness ± SD at the subfoveal, superior, temporal, inferior, and nasal locations was 260 ± 84, 248 ± 77, 219 ± 57, 197 ± 65, and 158 ± 75 μm, respectively. All differences in macular choroidal thickness were statistically significant, except for the difference between superior and subfoveal measurements (p = 0.604) (Table 1).

**Table 1:** Macular Choroidal Thickness Differences (p-values shown)

p-Value	Subfoveal	Superior	Temporal	Inferior
Superior	0.604	--	--	--
Temporal	0.003	0.022	--	--
Inferior	<0.001	<0.001	0.007	--
Nasal	<0.001	<0.001	<0.001	<0.001

**Fig. 2:**
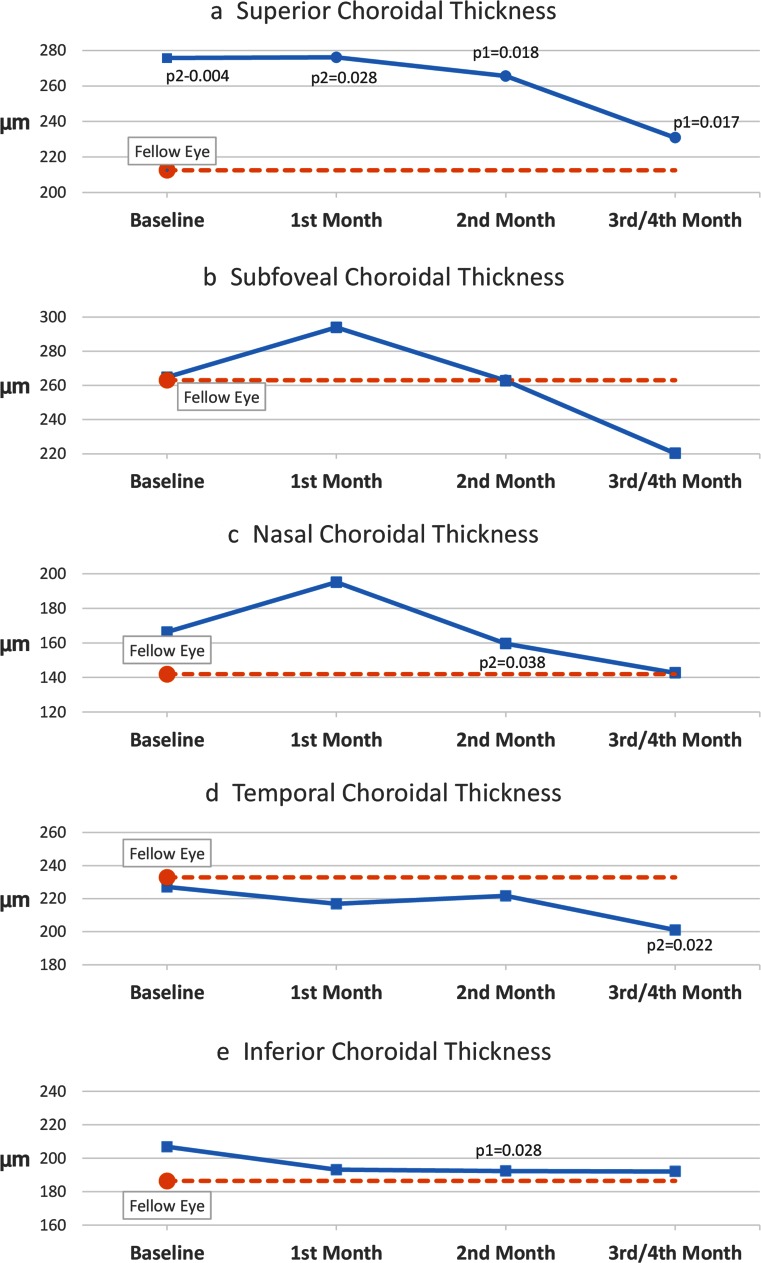
Macular choroidal thickness measurements (**a** Superior, **b** Subfoveal, c Nasal, **d** Temporal, **e** Inferior). Only significant p-values are shown. p1, p-value for comparison with baseline measurements in the study eye. p2, p-value for comparison with fellow eye measurements

Macular choroidal thickness measurements are shown in Table 2. Superior macular choroidal thickness in the study eye was significantly larger at baseline and 1st month exams (276 ± 74 and 276 ± 84 μm, respectively) when compared with the control eye (213 ± 82 μm) (p = 0.004 and p = 0.028, respectively). Thickness significantly decreased at the 2nd month and 3rd/4th month follow-up exams to 266 ± 79 and 231 ± 40 μm, respectively (compared with baseline thickness in the study eye (p = 0.018 and p = 0.017, respectively) (Fig. 2a).

**Table 2:** Macular Choroidal Thickness Changes

Measured Location			Mean (μm)	STD^a^ (μm)	p-Value	
					Compared with Control Eye	Compared with Study Eye Baseline
Subfoveal	Control (Fellow) Eye		263.04	87.79	–	–
	Study Eye	Baseline ^b^	264.83	84.47	0.638	–
		Month 1	293.93	94.82	0.612	0.395
		Month 2	262.78	84.51	0.515	0.058
		Months 3-4	220.25	47.79	0.508	0.373
Nasal	Control (Fellow) Eye		141.92	62.75	–	–
	Study Eye	Baseline ^b^	166.33	80.68	0.347	–
		Month 1	195.14	87.20	0.128	0.271
		Month 2	159.56	79.74	0.038*	0.735
		Months 3-4	142.64	54.47	0.424	0.575
Superior	Control (Fellow) Eye		212.54	81.61	–	–
	Study Eye	Baseline ^b^	275.73	74.20	0.004*	–
		Month 1	276.14	84.45	0.028*	0.398
		Month 2	265.63	79.33	0.123	0.018*
		Months 3-4	230.83	40.08	0.110	0.017*
Temporal	Control (Fellow) Eye		232.85	59.60	–	–
	Study Eye	Baseline ^b^	227.09	50.63	0.722	–
		Month 1	216.86	53.56	0.735	0.753
		Month 2	221.67	71.48	0.314	0.398
		Months 3-4	201.00	52.32	0.022*	0.093
Inferior	Control (Fellow) Eye		186.40	77.23	–	–
	Study Eye	Baseline ^b^	206.83	56.93	0.158	–
		Month 1	193.14	66.50	0.237	0.612
		Month 2	192.33	66.35	0.214	0.028*
		Months 3-4	192.10	58.06	0.241	0.327

^a^ STD = Standard Deviation

^b^ Baseline = Measurement on the day of TASS diagnosis

* Statistically Significant

Subfoveal and nasal macular choroidal thickness increased at baseline and 1st month exams and decreased at the 2nd and 3rd/4th month exams. These differences were not statistically significant (both for comparison with the control eye and comparison with baseline thickness in the study eye). Only one significant difference was shown in nasal choroidal thickness between study and controls eye at the 2nd month exam (see Fig. 2b and c). No specific pattern was shown in temporal and inferior macular thickness (see Fig. 2d and e).

### Peripapillary choroidal thickness measurements

Mean peripapillary choroidal thickness ± SD at the superior, temporal, nasal, and inferior locations was 181 ± 63, 170 ± 74, 150 ± ,60 and 128 ± 53 μm, respectively. All differences between peripapillary choroidal thickness locations were statistically significant (p < 0.001), except for the difference between temporal and nasal measurements (p = 0.081).

**Fig. 3:**
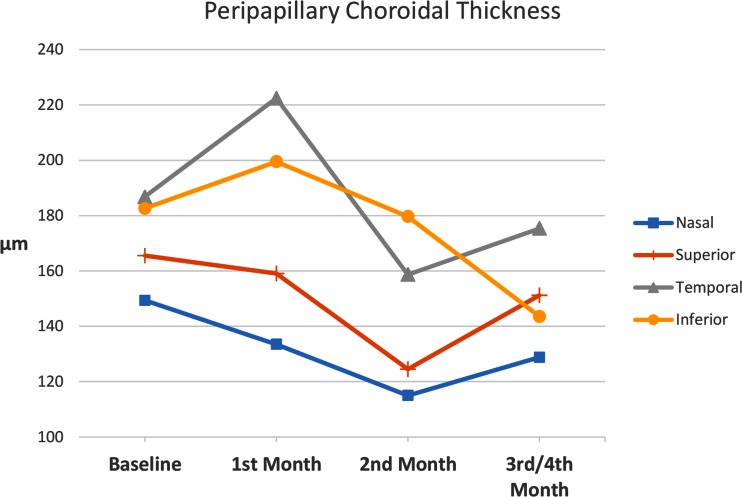
Peripapillary choroidal thickness measurements

Peripapillary choroidal thickness measurements are shown in Table 3. Superior, temporal, nasal, and inferior baseline thickness in the study eye were 187 ± 61, 183 ± 84, 166 ± 71, and 149 ± 61 μm, respectively. These were larger than control eye measurements which were 180 ± 66, 151 ± 63, 143 ± 56, and 116 ± 59 μm, respectively. The differences were statistically significant for the nasal (p = 0.026) and inferior (p = 0.033) locations. In the superior (187 ± 61 μm) and nasal (166 ± 71 μm) locations maximal thickness was at the baseline exam. In the temporal (183 ± 84 μm) and inferior (149 ± 61 μm) locations maximal thickness was at the 1st month exam. These differences were not statistically significant (Table 3). Peripapillary thickness decreased thereafter (Fig. 3). Differences were not statistically significant when compared with baseline measurements in the study eye (Table 3).

**Table 3:** Peripapillary Choroidal Thickness Changes

Measured Location			Mean (μm)	STD^a^ (μm)	p-Value	
					Compared with Control Eye	Compared with Study Eye Baseline
Inferior	Control (Fellow) Eye		116.19	58.66	–	–
	Study Eye	Baseline ^b^	149.36	60.74	0.033*	–
		Month 1	133.50	54.66	0.133	0.058
		Month 2	115.00	49.62	0.917	0.144
		Months 3-4	128.80	55.38	0.285	0.173
Nasal	Control (Fellow) Eye		142.94	56.41	–	–
	Study Eye	Baseline ^b^	165.55	71.22	0.026*	–
		Month 1	159.08	68.74	0.046*	0.833
		Month 2	124.50	49.40	0.917	0.273
		Months 3-4	151.20	70.69	0.508	0.066
Superior	Control (Fellow) Eye		180.03	66.11	–	–
	Study Eye	Baseline ^b^	186.82	60.63	0.790	–
		Month 1	222.40	42.40	0.345	0.891
		Month 2	158.67	78.07	0.917	0.080
		Months 3-4	175.35	60.25	0.959	0.779
Temporal	Control (Fellow) Eye		151.65	62.53	–	–
	Study Eye	Baseline ^b^	182.60	84.15	0.386	–
		Month 1	199.50	107.75	0.249	0.414
		Month 2	179.67	89.20	0.028*	0.500
		Months 3-4	143.55	44.06	0.959	0.726

^a^ STD = Standard Deviation

^b^ Baseline = Measurement on the day of TASS diagnosis

* Statistically Significant

### Central retinal thickness and nerve fiber layer measurements

**Fig. 4:**
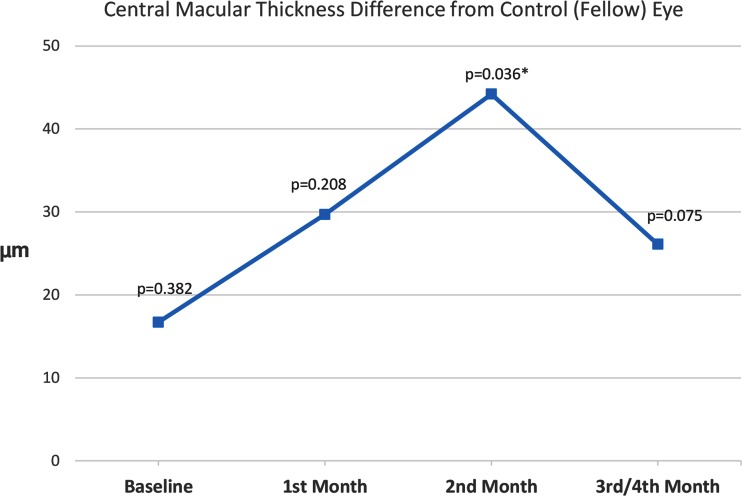
Central macular thickness difference from control (fellow) eye. *Significant p-values; p-values are for comparison with fellow eye measurements

Mean CMT at baseline was 288 ± 48 μm, compared with 271 ± 38 μm in the control (fellow) eye. This difference of 17 μm was not statistically significant (p = 0.382). Changes in CMT at the 1st, 2nd, and 3rd/4th month exams are shown in Fig. 4. Difference in CMT between the study and control eyes was statistically significant only at the 2nd month exam (a difference of 44.2 μm, p = 0.036) (Fig. 4).

Peripapillary NFL thickness did not change significantly in any location and showed no specific pattern (Table 4).

**Table 4:** Nerve fiber layer thickness changes

Location		Mean (μm)	STD^a^ (μm)	p-Value*
Nasal	Baseline ^b^	88.18	34.19	–
	Month 1	92.50	45.11	0.246
	Month 2	79.30	11.79	0.893
	Months 3-4	92.70	39.80	0.573
Superior	Baseline ^b^	124.36	34.40	–
	Month 1	122.42	38.00	0.753
	Month 2	114.33	38.72	0.138
	Months 3-4	123.15	24.39	0.086
Temporal	Baseline ^b^	71.09	17.68	–
	Month 1	73.92	22.32	0.596
	Month 2	70.50	16.02	0.715
	Months 3-4	67.30	12.07	0.528
Inferior	Baseline ^b^	123.00	30.11	–
	Month 1	107.17	22.73	0.600
	Month 2	118.67	31.77	0.686
	Months 3-4	128.80	20.16	0.141

^a^ STD = Standard Deviation

^b^ Baseline = Measurement on the day of TASS diagnosis

* p-Values represent comparisons with Baseline measurements

## Discussion

In this study, we evaluated quantitatively possible changes in choroidal, retinal, and nerve fiber layer thickness following an occurrence of TASS. Few studies have used EDI-OCT to evaluate inflammatory-related changes in choroidal thickness. Published studies evaluated diseases with a known chorioretinal involvement, such as Vogt-Koyanagi-Harada [12, 15]. and Multiple Evanescent White Dot Syndrome (MEWDS) [16]. We attempted to discover subtle inflammatory changes in the choroid, resulting from an acute anterior segment inflammatory process, with no clinical evidence of posterior segment involvement.

Macular choroidal thickness in the superior, subfoveal, and nasal macula did increase and was larger than the fellow eye. This was statistically significant only for the superior macula. Peripapillary choroidal thickness was larger in the study eye at baseline compared with the control eye. This difference was statistically significant for the nasal (p = 0.026) and inferior (p = 0.033) locations. There appeared to be a pattern in all peripapillary choroidal locations, showing a peak thickness at the baseline or 1st month exam with a decrease thereafter. However, these changes were not statistically significant. There are no previous publications examining choroidal thickness changes following TASS. Two recent publications examined changes in subfoveal choroidal thickness following uneventful cataract extraction and had conflicting results. The first paper found a significant increase in subfoveal choroidal thickness following cataract surgery in 29 patients [17]. The second paper analyzed both subfoveal and peripapillary choroidal thickness measurements and found no significant change following cataract extraction [18]. Therefore, the trend found in our study may be attributed to the TASS, but evidence in the literature regarding choroidal changes following cataract extraction are inconclusive.

We also observed constant significant differences in choroidal thickness between different measurement locations. In the macular region, thickness ratio was subfoveal > superior > temporal > inferior > nasal (260 ± 84, 248 ± 77, 219 ± 57, 197 ± 65, and 158 ± 75 μm, respectively). These differences are consistent with previously published data and include nasal macular choroid is the thinnest [10, 19], superior macular choroid is thicker than inferior macular choroid [13, 14], subfoveal choroid is thicker than either nasal or temporal macular choroid [11,14,19]. Also, mean subfoveal choroidal thickness in our study (260 ± 84 μm) is similar to the value found in a recently published, large population-based study, performed on 3,233 subjects (254 ± 107 μm) [20]. In the peripapillary region, thickness ratio was superior > temporal > nasal > inferior (181 ± 63, 170 ± 74, 150 ± 60, and 128 ± 53 μm, respectively). Previous literature shows that superior peripapillary choroid is thicker than inferior peripapillary choroid [21–23]. The correlation of our findings with previously published data strengthens the validity of our measurements and vice versa.

Measurements of retinal CMT showed an increase in retinal thickness, peaking at the 2nd month (statistically significant) and decreasing thereafter. The changes can be attributed to a transient subclinical macular inflammatory response to the cataract procedure and not to the TASS itself. Such changes following cataract surgery have been previously demonstrated in the literature [18, 24, 25]. Two patients developed CME during follow-up. In both cases, CME was not evident at the 2nd month follow-up and appeared later. Therefore, it did not seem to be a major contributor to the peak CMT change seen at month 2. No significant change in NFL thickness was observed.

In conclusion, TASS may have a transient effect on the choroid. Retinal changes observed may be related to the cataract procedure itself as previously described. We acknowledge some limitations of the study, which include a small sample size and a lack of OCT measurements prior to the occurrence of TASS. Choroidal measurements were corrected for inter-eye axial length differences, but compensating for the known diurnal variation in choroidal thickness [26, 27] was not feasible in the outpatient clinic setting.

The novel use of EDI-OCT enabled accurate imaging and quantification of choroidal changes. This study demonstrated that a significant inflammatory reaction involving the anterior uvea, such as TASS, might also have an effect on the posterior uvea. Further studies are needed to corroborate this. The continued use of EDI-OCT will help prove or refute theories of choroidal involvement in different pathologies and provide better understanding of disease processes and etiologies.
